# Enhancing Photostability of Prochloraz via Designing Natural Acid-Derived Prochloraz-Based Ionic Liquids

**DOI:** 10.3390/molecules30071641

**Published:** 2025-04-07

**Authors:** Zhiqiang Gao, Fengmao Liu, Qingrong Peng, Wenzhuo Wang

**Affiliations:** 1 Innovation Center of Pesticide Research, Department of Applied Chemistry, College of Science, China Agricultural University, Beijing 100193, Chinaqrpeng@cau.edu.cn (Q.P.); 2 Institute for the Control of the Agrichemicals, Ministry of Agriculture and Rural Affairs, Beijing 100125, China

**Keywords:** prochloraz, ionic liquids (ILs), physicochemical properties, photostability, fungicide

## Abstract

Converting pesticides into ionic liquids by designing counterions can modulate their physicochemical properties, thus improving their efficacy and environmental safety. In this study, eight prochloraz-based ionic liquids (PILs) were synthesized using natural organic acids, and their physicochemical properties, toxicity, antifungal activity, and efficacy in postharvest mango preservation were evaluated. The results showed that the physicochemical properties of propiconazole, including water solubility, logK_ow_, surface activity, and light stability, could be adjusted by selecting counterions with varying structures. These properties were correlated with toxicity to zebrafish embryos and antifungal activity against *Colletotrichum gloeosporioides*. Notably, except for the benzoate PIL, the photostability of the other seven PILs was enhanced under UV irradiation, with the cinnamate PIL exhibiting a half-life 2.28 times longer than prochloraz. Spectral analysis indicated that the anions influenced photostability by shielding or interacting with the cations. Furthermore, the three selected PILs improved pesticide deposition on the mango surface during preservation, and the salicylate PIL enhanced pesticide penetration into the fruit, potentially contributing to its therapeutic activity. In conclusion, the ionic liquid strategy offers an effective method to modify pesticide properties, improve photostability, reduce losses, and optimize pesticide formulation.

## 1. Introduction

Fungicides protect plants from fungal diseases, ensure healthy crop growth, and maintain food safety [1,2]. However, the efficacy of some photo-unstable pesticides is hindered by their physicochemical properties and suboptimal formulations, resulting in significant pesticide losses and low utilization [3,4,5]. Researchers have conducted numerous studies on pesticide modification and formulation optimization to improve pesticide photostability and efficacy [6,7,8].

Ionic liquids (ILs) are roughly defined as salts that melt below 100 °C. By choosing appropriate ion candidates to meet particular functionality needs, ILs’ physicochemical and biological properties can be readily adjusted, offering an easy and potentially promising way to modify compound properties [9,10,11]. Transforming active pharmaceutical ingredients (APIs) into ionic liquids (API-ILs) has demonstrated significant promise for drug delivery by addressing polymorphism, customizing solubility, enhancing thermal stability, augmenting dissolution, regulating drug release, adjusting surfactant characteristics, boosting API permeability, and modifying the biological activity of active compounds [12,13,14]. Numerous API-ILs have been documented based on agricultural chemicals such as MCPA, dicamba, chlormequat chloride, glyphosate, picloram, nicosulfuron, triflumizole, imazalil, tebuconazole, and pyrimethanil. Such studies have shown that transforming agrochemicals into ionic liquid can improve key properties, including biological activity, rain leaching resistance, photostability, and reduced volatilization and leaching from soils, compared to the original compounds [15,16,17,18,19,20]. Moreover, environmental studies suggest that herbicidal ionic liquids persist in the environment as mixtures of common ions without contributing additional environmental burdens as a new type of contaminant [21,22,23,24]. These findings underscore the promising potential of pesticide ionic liquids to optimize formulations and improve the efficacy of pesticides.

Prochloraz (N-propyl-N-(2,4,6-trichlorophenoxy)ethyl-imidazole-1-carboxamide) is a broad-spectrum imidazole fungicide commonly applied as a foliar spray or fruit dip. It effectively controls a wide range of diseases caused by ascomycetes and hemipterans in various crops, including oilseeds, ornamentals, fruits, and vegetables. However, prochloraz is photo-unstable, with a photolysis half-life of 1.5 days in water, which may lead to substantial losses of the active ingredient during applications. Additionally, it exhibits moderate toxicity to aquatic organisms, with embryonic mortality and developmental effects observed at concentrations of 2.4 and 4.8 mg/L [25]. Therefore, developing new efficient and environmentally friendly pesticide products of prochloraz is important to promote efficient utilization and reduce the negative environmental impacts on fish.

Prochloraz is a weak base with a pKa value of 3.8 containing a free nitrogen atom and possesses the ability to accept protons, which could be converted to new prochloraz-based ionic liquids (PILs) by pairing with appropriate acids via acid–base reaction. This work investigates the physicochemical and biological properties of PILs with various anions derived from natural substances, including acetic, lactic, pyruvic, nonanoic, oleic, benzoic, salicylic, and cinnamic acids [26,27,28,29,30,31,32]. The PILs were prepared through a one-step reaction involving acids and prochloraz, followed by characterization using differential scanning calorimetry and ^1^H NMR. The hydrophilic–hydrophobic nature of the anions may influence the physicochemical properties of the PILs, potentially affecting their distribution, toxicity to non-target organisms, and bioactivity against pathogenic bacteria. As part of the investigation, the study discusses how different anions impact the photostability of the active ingredients. It also explores the solubility, surface activity, octanol-water partition coefficient, acute toxicity to fish embryos, and antifungal activity of the synthesized PILs. Furthermore, the research delves into the relationships between anion type, physicochemical properties, and application effects, providing valuable insights for designing and developing imidazole-based pesticide ionic liquids.

## 2. Results and Discussion

### 2.1. Preparation and Characterization of PILs

**Scheme 1:**
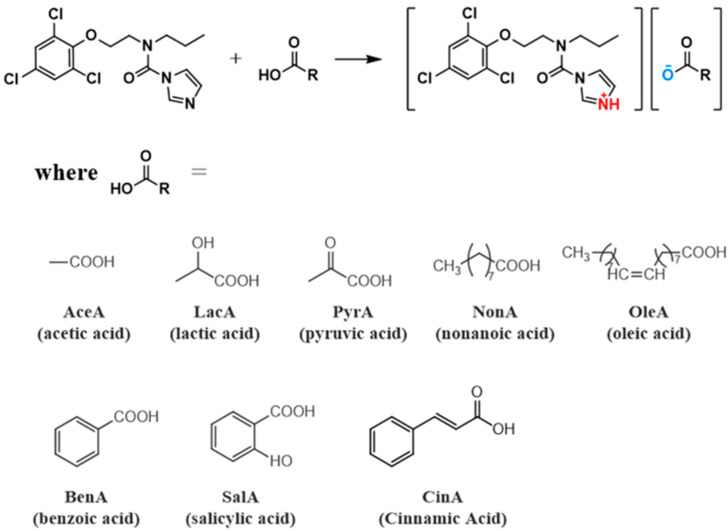
Synthesis of PILs and the structures of anions.

**Figure 1:**
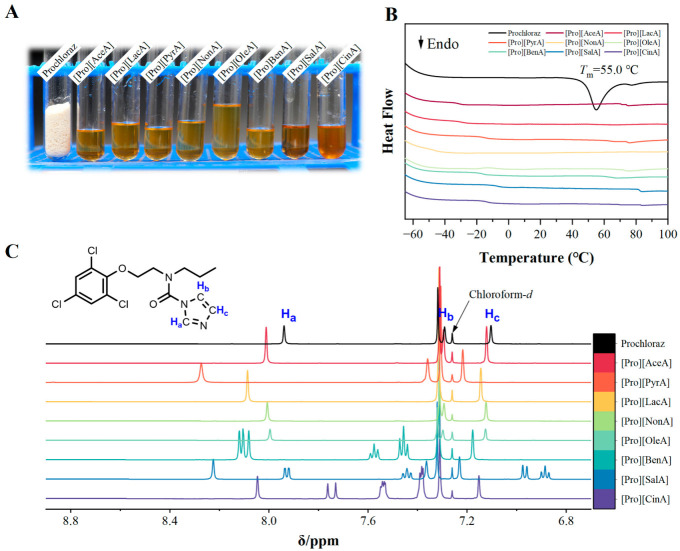
Characterization of prochloraz-based ionic liquids (PILs): state at room temperature (**A**), differential scanning calorimetry (**B**), and partial ^1^H NMR profiles (**C**).

Prochloraz-based ILs with different naturally occurring organic acids were synthesized by an acid–base reaction in methanol. Scheme 1 depicted the structures of the anions and the synthesis pathways of the PILs. The counterions were derived from a series of naturally occurring organic acids exhibiting diverse structural features, including hydrocarbons of varying chain lengths, hydrophilic groups, and aromatic rings. Specifically, these included acetic acid (AceA), lactic acid (LacA), pyruvic acid (PyrA), nonanoic acid (NonA), oleic acid (OleA), benzoic acid (BenA), salicylic acid (SalA), and cinnamic acid (CinA). As naturally sourced compounds, these acids typically degrade rapidly in the environment. Consequently, it will not cause additional environmental impacts when used to modify fungicides. According to the product characterization results in Figure 1, all PILs obtained were liquid at room temperature. The purities of the obtained PILs confirmed by HPLC-UV are above 95% (Table S1). The Differential scanning calorimetry (DSC) spectra (Figure 1B) showed that the products did not show any heat absorption peaks near 55 °C, thus demonstrating the creation of a new microsystem. In addition, ^1^H NMR spectroscopy was used to characterize the prepared PILs; the chemical shift of hydrogen on the imidazole ring to the lower field proved the interaction between prochloraz and the anions. The detailed ^1^H NMR data are presented in Table S2 and the Supporting Information.

### 2.2. Physicochemical Properties

#### 2.2.1. Solubility

The solubility of the prepared PILs investigated in six solvents varying from high to low polarity is shown in Table 1. The synthesized PILs exhibited different solubility compared to prochloraz in ethyl acetate, toluene, and hexane. Additionally, the PILs demonstrated good solubility in methanol and acetone, but their solubility in water was relatively low, with no significant difference observed.

**Figure 2:**
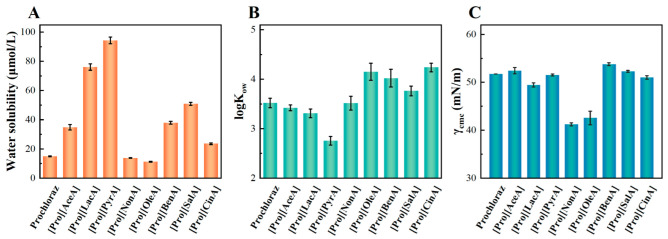
Water solubility (**A**), the octanol-water partitions coefficients (**B**), and the interfacial surface tensions (**C**) of prochloraz and PILs at 25 °C.

In water solubility experiments, no dissociation or solid precipitation was observed (neutral prochloraz as solid precipitates) when the ionic liquids were shaken with water for 48 h. This demonstrates that the ionic liquids remain stable when in contact with water. The results of the aqueous solubility measurements of the PILs are shown in Figure 2A. It can be seen that the introduction of different paired ions has an increasing or decreasing effect on the solubility of the active ingredient, which is strongly correlated with the aqueous affinity of the paired ions. The solubility of [Pro][AceA], [Pro][LacA], and [Pro][PyrA] increased with the increase in hydrophilic functional groups, and the solubility of [Pro][NonA] and [Pro][OleA] slightly decreased with the rise in the carbon chain length, and the hydrophobicity increased. These results, consistent with previous studies, demonstrated the effectiveness of converting compounds to ionic liquids in altering their solubility [33,34]. However, this variation in water solubility is insufficient to dissolve compounds directly into water for spray application. Therefore, the use of PILs requires further formulation development studies.

#### 2.2.2. Octanol-Water Partition Coefficient

In this study, prochloraz was transformed into eight imidazolium-based ionic liquids by matching different anions, with logK_ow_ from 3.52 to 2.75–4.23 (Figure 2B). This variation is closely linked to the hydrophilicity of the anions, demonstrating the potential of IL design to mitigate environmental risks while improving the efficacy of prochloraz. Previous studies have similarly shown that ionic liquids with appropriate octanol-water partition coefficients can be synthesized by selecting suitable paired ions, which, in turn, help reduce the environmental risks associated with pesticides [35].

The octanol-water partition coefficient reflects the distribution of pesticides between organic and aqueous phases, providing important guidance on pesticide biological activity, non-target toxicity, deposition and migration, and environmental behavior [36,37]. A further comprehensive study of the relation between logK_ow_ of PILs and fungicidal efficiency, non-target biotoxicity, and ecological effects is of great significance for the purposeful design of PILs for effective and improved pesticide applications.

#### 2.2.3. Surface Activity

As indicated in Figure 2C, it can be concluded that the surface activity of different acid-derived PILs does not vary significantly, except for the anion with surface properties. [Pro][NonA] (41.24) and [Pro][OleA] (42.53) showed a significant reduction in surface tension compared to prochloraz (51.75). This reduction is likely due to the long alkyl chain in the anion, which positively affects surface properties. The findings were similar to those from previous studies [38,39].

It is common for a spray solution to have low surface tension to increase the retention time and absorption of active ingredients in the plant [17]. Modifications of the surface activity of compounds may also influence the use of surfactants in the formulation, dispersion, and retention of active ingredients on the leaf or fruit surface and even enhance biological activity.

### 2.3. Photostability and Its Mechanism Analysis

**Figure 3:**
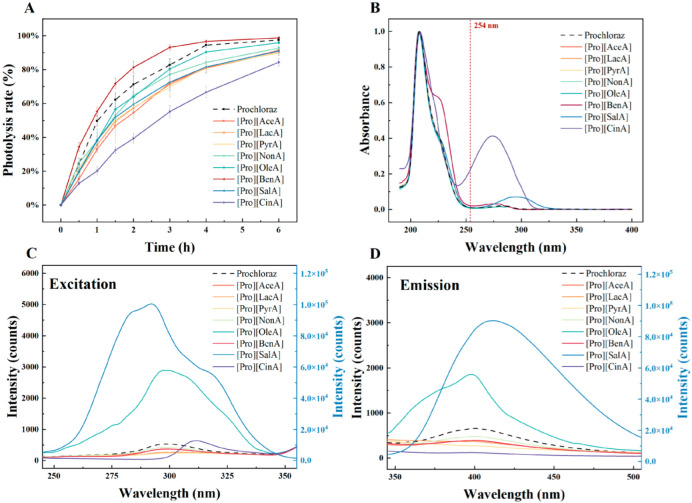
The photolytic curves under UV radiation (**A**), UV absorption spectrum (**B**), excitation spectra (**C**), and emission spectra (**D**) of prochloraz and PILs.

Table 2 and Figure 3A showed the stability of prochloraz and the obtained PIL under UV irradiation at 25 °C. The results showed that the photolysis half-lives of most PILs were more prolonged than that of prochloraz (1.08 h), ranging from 1.26 to 2.27 h, except for [Pro][SalA] (0.90 h), which had a slightly shorter half-life than prochloraz. Generally, longer half-lives contribute to reduced ineffective degradation of the active ingredient on the plant surface, thereby increasing pesticide effectiveness. Notably, [Pro][CinA] had a photodegradation half-life 2.28 times longer than prochloraz, surpassing results from some previous studies on microencapsulation dosage form design, and merits further attention [40,41]. This strategy may facilitate the development of new photostabilized prochloraz formulations, potentially increasing the effective application rate of the pesticide.

In this paper, the photostability mechanism was explored by UV and fluorescence spectroscopic studies. On the one hand, the counterions in the PILs can reduce the absorption of photon energy by the compound or reduce the absorption of energy that leads to the cleavage of the compound’s covalent bonds. The excitation spectra (Figure 3C) showed that except for [Pro][OleA], [Pro][SalA], and [Pro][CinA], the remaining PILs have fluorescence emission and excitation behaviors similar to those of prochloraz. The excitation spectral signal intensities are slightly weaker than those of prochloraz in the same concentration. This represented the absorption of less photon energy in favor of the photostability of the compounds. Although the excitation peak signals of [Pro][OleA] and [Pro][SalA] are more potent than that of prochloraz, the emission spectra (Figure 3D) of both of them have correspondingly stronger signal peaks, which represents that they can release the energy of the excited state by increasing the release of fluorescence, avoiding the accumulation of molecular energy in the excited state that leads to the breakage of covalent bonds and degradation.

On the other hand, the introduction of anions can provide some light-shielding effects at the same time. Figure 3B illustrates the UV absorption spectra of different ionic liquids where the introduction of benzoate, salicylate, and cinnamate anions with aromatic ring structures increases the absorbance and may lead to some UV shielding. Such UV shielding effect may be one of the reasons for the increase in the photostability of [Pro][SalA] and [Pro][CinA] [7,41]. In addition, the excitation spectrum of [Pro][CinA] almost disappeared the signal at 299 nm, and instead, a new peak with similar intensity to that of prochloraz appears near 312 nm. At the same time, [Pro][CinA] did not observe a significant signal in the emission spectrum. This may indicate that the cinnamate anion, acting as a photo-masking agent, absorbed photon energy before absorbing prochloraz cation and degraded during exposure of the [Pro][CinA] solution to light. A comparative photostability study indicated that [Pro][CinA] exerts a more substantial stabilizing effect for prochloraz than the mixture of prochloraz and sodium cinnamate (1.89 h, shown in Table S3) at the corresponding concentration, likely due to specific interactions and a more favorable chemical environment that protects against photodegradation. This also demonstrated that both the ionic effect of prochloraz and the photomasking effect of the anion are responsible for the improved photostability of PILs.

### 2.4. Acute Toxicity to Zebrafish Embryo

**Figure 4:**
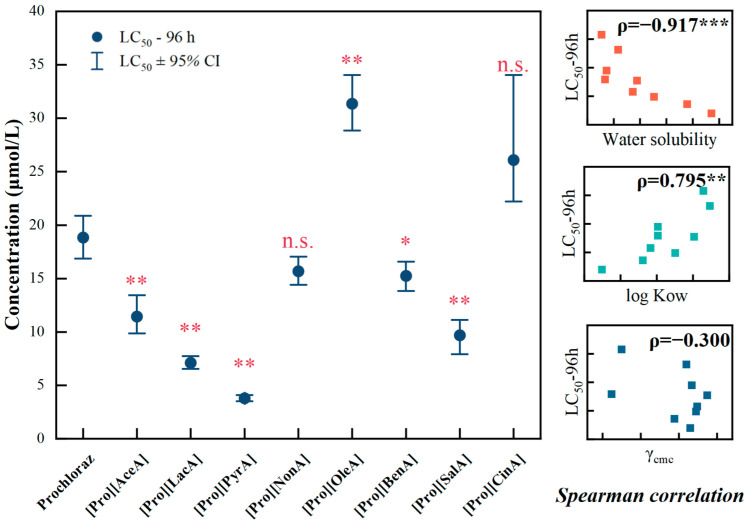
Acute toxicity of prochloraz and PILs to zebrafish embryo (significance vs. prochloraz, red) and Spearman correlation of LC_50_-96 h with physicochemical properties. Significance levels: n.s. *p* > 0.05, * *p* < 0.05, ** *p* < 0.01, *** *p* < 0.001.

The 96 h LC_50_ values and 95% confidence intervals (CIs) exposed to prochloraz and the PILs with various anions are presented in Figure 4 and Table S4. Lower LC_50_ values indicate that the compounds were more toxic to zebrafish embryos. Consistent with previous studies, the 96 h LC_50_ of prochloraz for zebrafish embryos was 7.09 mg/L, indicating moderate toxicity [42]. The toxicity of PILs to zebrafish embryos varied, with the order of toxicity as follows: [Pro][PyrA] > [Pro][LacA] > [Pro][SalA] > [Pro][AceA] > [Pro][BenA] > [Pro][NonA] > Prochloraz > [Pro][CinA] > [Pro][OleA]. The correlation of LC_50_-96h with water solubility, logK_ow_, and γ_cmc_ was evaluated by Spearman rank correlation evaluation, and the results are shown in Figure 4. For PILs, the LC_50_-96 h to zebrafish embryos showed a significant correlation with water solubility (|ρ| > 0.81) and a strong correlation with logK_ow_ (0.61 < |ρ| < 0.80). It can be concluded that the design and preparation of PILs with low solubility may help to reduce the toxicity of prochloraz to aquatic organisms. Similarly, previous studies have shown that preparing triflumizole ionic liquids with natural anions reduced the toxicity to adult zebrafish. Therefore, designing and preparing active ingredient ionic liquids can help modify the toxic effects of compounds, potentially reducing the threat of prochloraz to the aquatic environment.

The latest research on the toxicity of pesticide ionic liquids to environmental organisms has provided new insights, suggesting that ionic liquids exist in the environment in their typical ionic form rather than a new type of contaminant [23,24]. This implies that ionic liquids in the environment may exist in forms other than the subject state in this paper. Since the counterions considered in this paper are natural compounds commonly used in agricultural applications, applying the PILs proposed here will unlikely result in additional environmental impacts.

### 2.5. Fungicidal Activity Against Colletotrichum Gloeosporioides

**Figure 5:**
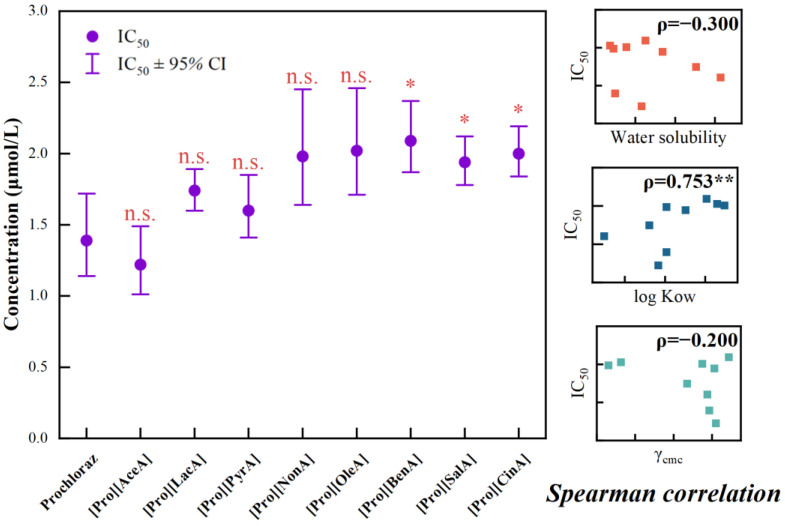
Fungicidal activity against *Colletotrichum gloeosporioides* of prochloraz and PILs (significance vs. prochloraz, red) and Spearman correlation of IC_50_ with physicochemical properties. Significance levels: n.s. *p* > 0.05, * *p* < 0.05, ** *p* < 0.01.

The toxicity regression equations and inhibitory concentration (IC_50_) of prochloraz and PILs against *Colletotrichum gloeosporioides* are shown in Figure 5 and Table S5. The IC_50_ values and their 95% confidence intervals indicate variations in the bioactivity of different PILs. The bioactivity of [Pro][AceA], [Pro][LacA], [Pro][PyrA], and [Pro][NonA] did not significantly differ from that of prochloraz. Moreover, [Pro][OleA], [Pro][BenA], [Pro][SalA], and [Pro][CinA] exhibited slightly reduced bioactivity compared to prochloraz. The IC_50_ showed a strong correlation with logK_ow_ (0.61 < |ρ| < 0.80), which may be related to the fact that lipophilic anions help the cationic components to enter the fungal biofilm. Similarly, introducing anions has been shown to affect the activity of compounds against a wide range of fungal diseases [39,43]. When an anion is introduced into an antimicrobial compound, it forms an organic salt that retains its antibacterial activity while exhibiting enhanced biological activity or a broader spectrum of activity due to the synergistic interaction between the anion and the cation.

Furthermore, a comparative fungicidal activity of eight acids and their sodium salts (Table S6) indicated that the anion and the acid did not demonstrate a comparable fungicidal impact in PILs. These results showed that the cations (ionized fungicides) played a significant role in inhibiting the fungal growthand that the type of anion had little significant effect.

In conclusion, modification by conversion to ionic liquids did not significantly alter the fungicidal activity against *Colletotrichum gloeosporioides*, providing some assurance for the practical application of PILs.

### 2.6. Pesticide Distribution of Postharvest Mangoes

Postharvest preservation technology is a technique to prevent and control postharvest diseases through postharvest fruit dipping and storage, which is widely used in fruit and vegetable agricultural products. In this study, we used prochloraz and three kinds of PILs to configure the medicinal solution for the postharvest fruit dipping treatment of mango to observe the distribution of pesticides after dipping and even during the storage process.

**Figure 6:**
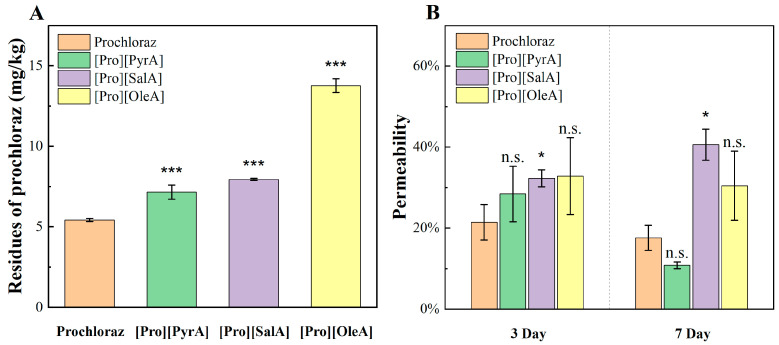
Initial deposition (**A**) and permeability (**B**) of active ingredients after postharvest treatment of mango by prochloraz, [Pro][PyrA], [Pro][SalA], and [Pro][OleA]. Significance levels: n.s. *p* > 0.05, * *p* < 0.05, *** *p* < 0.001.

Prochloraz on mango, as shown in Figure 6A, after PILs dipping, the content of the active ingredient on the fruit was significantly greater than that of the prochloraz treatment group. Three ionic liquids were conducive to increased deposition of the active ingredient on fruit, which was more conducive to full use of the active ingredient [44]. Figure 6B shows the difference in the permeability of the mango interior on the third day and the seventh day. Overall, the penetration of both prochloraz and PILs into the interior of the mango epidermis was poor, with the penetration rates of the different treatments being less than 0.5% on the seventh day. Comparatively, the penetration rates of the [Pro][SalA] treatment group were slightly higher than those of the prochloraz treatment group on the third and seventh days, with some significance. This might be caused by the fact that salicylic acid, as a plant resistance elicitor, stimulated the uptake and metabolism of exogenous compounds in the mango organism [45]. These findings demonstrate the potential application of PILs in fruit preservation.

## 3. Materials and Methods

### 3.1. Materials

Prochloraz (99%) was purchased from Hubei Jiahui Xingcheng Biotechnology Co., Ltd. (Wuhan, China). Methanol, acetone, toluene, hexane, 1-octanol, acetic acid, lactic acid, pyruvic acid, nonanoic acid, oleic acid, benzoic acid, salicylic acid, and cinnamic acid were analytical grade reagents and purchased from Sinopharm Chemical Reagent Co., Ltd. (Beijing, China). Acetonitrile was high-performance liquid chromatography (HPLC) grade and supplied from MREDA Chemical and Biological Regent Co., Ltd. (Beijing, China). *Colletotrichum gloeosporioides* (ACCC 36431) was collected and provided by the Chinese Agricultural Microbial Strain Collection and Management Centre (ACCC).

### 3.2. Preparation and Characterization of PILs

PILs were prepared using the procedure outlined in the literature, which involved adding 30 mL of methanol to a round-bottom flask along with prochloraz (0.001 mol) and the same stoichiometric amount of organic acid [39,46]. The mixture was stirred at 50 °C for 3 h, followed by the evaporation of methanol through decompression distillation at 45 °C for 0.5 h, yielding the target product. ^1^H NMR spectra were acquired at 25 °C using a Bruker Avance DPX 500 MHz NMR spectrometer (Bruker, Berlin, Germany). The differential scanning calorimetry analysis of prochloraz and eight prepared PILs was conducted using a Hitachi DSC200, calibrated across a temperature range of −80 °C to 100 °C. Each sample was subjected to a heating rate of 10 °C/min and analyzed under a nitrogen atmosphere to minimize oxidative reactions and ensure inert conditions throughout the measurement process.

### 3.3. Determination of Physicochemical Properties

#### 3.3.1. Solubility

Following a widely used solubility procedure, the solubilities of prochloraz and all PILs in six solvents were determined [33]. These typical solvents were selected—water, methanol, acetone, toluene, ethyl acetate, and hexane. A 0.1 g sample of every chemical was weighed and added to a certain amount of solvent. Four types of behavior were characterized based on the dosage of the solvent used: high solubility, medium solubility, low solubility, and insoluble, which apply to chemicals that dissolved in 1, 2, and 3 mL of the solvent, respectively, and did not dissolve in 3 mL of the solvent. Every treatment was administered at 25 °C. The solubility in water was further determined using the following procedure: approximately 10 mg of prochloraz or PILs were weighed and added to 10 mL of distilled water. The mixture was shaken for 24 h, centrifuged, and filtered through a 0.22 μm membrane to obtain the saturated solution. All procedures were conducted in triplicate. The solubility in water was then measured using HPLC-UV. Instrument specifications are detailed in the Supporting Information.

#### 3.3.2. Octanol-Water Partition Coefficient

The partition coefficients (n-octanol/water) of prochloraz and the prepared PILs were estimated by the shake-flask method according to OECD guidelines (Test No. 117) [47]. The equilibrium partitioning of prochloraz or PILs between water and n-octanol was established, pre-saturated with each other. Initially, a n-octanol solution containing 2000 mg/L of the compound was mixed with water in centrifuge tubes at 1:2, 1:1, and 2:1 volume ratios, with two parallel samples for each ratio. All tubes were shaken at a constant temperature of 25 °C for 3 h. Subsequently, the samples were centrifuged at 4000 rpm for 10 min. The octanol and aqueous phases were then carefully collected and diluted tenfold with methanol, and their concentrations were measured using HPLC-UV and HPLC-MS/MS, respectively. Instrument specifications are detailed in the Supporting Information.

#### 3.3.3. Surface Activity

Through the Wilhelmy plate method, the interfacial surface tension of prochloraz and the produced PILs was measured at 25 °C using a JK 99B analyzer (Powereach, China) with a resolution of less than 0.05 mN/m [48]. The test solution (saturated aqueous solution) was prepared by dissolving approximately 0.05 g of prochloraz or PILs in 100 mL purified water under continuous agitation for 48 h at room temperature. Pure water’s surface tension was used to calibrate the device. After cleaning and drying, the sensing platinum plate was slowly oriented perpendicular to the solution interface. The plate will stop when the liquid surface tension and other relevant forces reach a balance. The interfacial surface tension values of the test chemicals were noted. Each compound was determined with three parallel samples.

### 3.4. Photostability and Mechanism Analysis

Photostability evaluation. Photostability studies of prochloraz and PILs were conducted at room temperature under UV exposure. Aqueous solutions containing each compound (at a concentration of 26.6 μmol/L) were prepared and hermetically sealed in quartz bottles. The bottles were then exposed to a 15 W UV lamp (Emax = 254 nm) at a distance of 15 cm, with three parallel samples for each compound. At different time intervals (0, 0.5, 1, 1.5, 2, 3, 4, and 6 h), 100 μL of the solutions was accurately removed from the vials, diluted tenfold with methanol, and analyzed by HPLC-MS/MS. Instrument specifications are detailed in the Supporting Information.

As dark controls, aluminum foil-wrapped quartz tubes were used to prevent the impact of outside variables. A first-order kinetic dissipation model was applied to the light stability data of prochloraz and PILs:

(1) $$C_{t}=C_{0}\times e^{-Kt},$$

where *C_t_* and *C*_0_ (mg/L) are the concentrations at *t* hour and zero hours, respectively, and *K* (h^−1^) is the first-order dissipation rate constant. The coefficient of determination r^2^ was used to describe the goodness of fit of the curves. The half-lives *t*_1/2_ of the chemicals were calculated by the following formula:

(2) t1/2=−ln⁡(0.5)K

UV–Vis Absorption Spectroscopy. The UV–Vis absorption spectra were acquired using a UV-1800PC spectrophotometer (Mapada, Shanghai, China). Before measurement, a sample solution was prepared by dissolving the compound of interest in methanol to a 2 mg/L concentration. A wavelength range of 190 nm to 400 nm was selected to cover the expected absorption spectrum of the sample. A blank solution, consisting of the same solvent used for the sample preparation, was placed in a quartz cuvette and utilized for baseline correction.

Fluorescence Spectroscopy. The fluorescence emission and excitation spectra were recorded using an RF-6000 fluorescence spectrometer (SHIMADZU, Kyoto, Japan). The sample was prepared by dissolving it in ethanol to achieve a 1 mmol/L concentration. For emission spectra, the samples were excited at 299 nm with both excitation and emission slit widths set to 1 nm, and the emission was scanned from 350 to 500 nm. For excitation spectra, the emission wavelength was fixed at 400 nm, and the excitation was scanned from 250 to 350 nm under the same slit width conditions. A blank measurement was performed using the solvent alone to correct for any background fluorescence. The sample solution was then placed into a clean quartz cuvette and introduced into the spectrometer.

### 3.5. Acute Toxicity to Zebrafish Embryo

The acute toxicity of prochloraz and PILs to fish embryos was evaluated according to OECD TG 236 [49]. Gradient solutions of the test substances were prepared at 1.3-fold concentration intervals using laboratory-oxygenated water. Appropriate Tween-80 was used to promote the dispersion of insoluble test substances in water. Thirty milliliters of each test solution were added to a 50 mL beaker, and ten well-developed embryos were transferred to each beaker. Three replicates were set up for each concentration. Dilution water controls and solvent controls were included to account for other potential influences.

During the exposure period, the embryos were maintained in an incubator at 26 ± 1 °C with a 14:10 h light/dark ratio. The test solutions were changed every 24 h, and the number of dead embryos was recorded at 24, 48, 72, and 96 h. Zebrafish embryo mortality was assessed based on egg coagulation, heart rate, and blood flow.

### 3.6. Fungicidal Activity Against Colletotrichum Gloeosporioides

The fungicidal activity of prochloraz and PILs was evaluated by conducting a growth inhibition assay on potato dextrose agar (PDA) plates [43]. Suspensions of the compounds at graded concentrations were added to molten PDA medium at approximately 50 °C to prepare media with different chemical concentrations (0.66, 1.33, 2.65, 5.31, 10.6, and 21.2 μmol/L). Pure water was used as a negative control. Mycelial plugs with a diameter of 5 mm were positioned in the middle of PDA plates that held various sample concentrations. Every treatment was carried out three times. Following a 96 h dark incubation period at 26 °C, each colony’s diameter was measured twice at right angles. The formula used to compute the suppression of mycelial growth was inhibition (%) = (*D_c_* − *D*_t_)/(*D_c_* − 5) × 100%, where *D_c_* and *D_t_* are the diameters in millimeters of the treatment colony and the control colony, respectively. Based on the acute toxicity and fungicidal activity data in the manuscript, approximate statistical tests were performed using known LC_50_/IC_50_ and their 95% confidence intervals. The tests, in turn, constructed t-statistics and determined the signifi-cance of differences between groups.

### 3.7. Pesticide Distribution of Postharvest Mangoes

The postharvest mango dipping procedure was modeled after the prochloraz EC formulation. Solutions with the same concentration of active ingredients were prepared by dispersing prochloraz and PIL separately in water containing an appropriate amount of Tween-80. Mangoes of uniform size were immersed in the solutions for 1 min, taken out, dried, and stored at 15 °C with 70% humidity, protected from light. At 0, 3, and 7 days, three to five mangoes were selected, peeled, pitted, and homogenized to obtain peel and pulp samples. Pesticides on the samples were detected by HPLC-MS/MS. Instrument specifications are detailed in the Supporting Information.

## 4. Conclusions

This study synthesized eight novel prochloraz-based ionic liquids (PILs) by utilizing several natural organic acids with different structures to modify the properties of prochloraz. Physicochemical and biological properties such as solubility, surface activity, octanol-water partitioning, photostability, acute toxicity to zebrafish embryos, fungicidal activity against *Colletotrichum gloeosporioides*, and pesticide distribution during postharvest preservation–storage on mango were studied.

The results showed that hydrophilic groups, such as hydroxyl and carbonyl, enhance the hydrophilicity of PILs, whereas anions with hydrophobic structures, such as long alkyl chains or benzene rings, contribute to liposolubility. Long alkyl chains also increase the surface activity of the compounds. The photostability of the active ingredients is increased, except for [Pro][BenA], by the shielding effect of the anions or by interactions between anions and cations. Notably, [Pro][CinA] increased photostability up to 2.28-fold. These findings demonstrate the potential of designing and improving the physicochemical properties of pesticides through the transformation into ionic liquids. Moreover, this also exhibited a potential application of IL-based improvement strategies for increasing the photostability of prochloraz and optimizing the formulation.

Spearman’s correlation analysis revealed a significant correlation between water solubility, logK_ow_, and toxicity towards zebrafish embryos and between logK_ow_ and fungicidal activity. It could be concluded that designing PILs with lower water solubility and higher logK_ow_ may reduce zebrafish embryos’ toxicity. However, further research is needed to ascertain the cause of the slight decline in inhibitory activity observed in PILs with higher logK_ow_. Additionally, the transition to PILs resulted in a higher deposition of the active ingredient on the fruit, while [Pro][SalA] improved its penetration into the fruit’s interior. This may improve the therapeutic bactericidal effect of prochloraz in postharvest preservation treatments of mangoes. Based on these findings, it appears that the transformation into ionic liquids can modify biological effects, environmental behavior, and the distribution of active ingredients in pesticides.

In summary, the IL-based improvement strategies proposed in this paper provide a practical method to modify the physicochemical properties of compounds. It offers promising applications in enhancing the photostability of prochloraz, mitigating toxicity to aquatic organisms, improving pesticide efficiency, and reducing negative environmental impacts. This work contributes valuable insights into pesticide formulation and efficacy optimization.

## Figures and Tables

**Table 1:** The solubilities of PILs in different solvents.

Substances	Solvents					
	Water 9.0 ^a^	Methanol 6.6 ^a^	Acetone 5.1 ^a^	Ethyl acetate 4.4 ^a^	Toluene 2.3 ^a^	Hexane 0.0 ^a^
Prochloraz	−	+	+	+	+	−
[Pro][AceA]	−	+	+	+	±	−
[Pro][LacA]	−	+	+	±	−	−
[Pro][PyrA]	−	+	+	±	−	−
[Pro][NonA]	−	+	+	+	+	−
[Pro][OleA]	−	+	+	+	+	+
[Pro][BenA]	−	+	+	+	+	−
[Pro][SalA]	−	+	+	+	+	−
[Pro][CinA]	−	+	+	+	+	−

^a^ Snyder polarity index. +: Good solubility. ±: limited solubility. −: poor solubility (<0.33 g/mL).

**Table 2:** The photolysis kinetics of prochloraz and PILs under UV radiation.

Chemicals	Regression Equation	$r^{2}$	K ($h^{-1}$)	$t_{1/2}$ (h)
Prochloraz	$C_{t}=10.2e^{-0.641t}$	0.997	0.641	1.08
[Pro][AceA]	$C_{t}=11.4e^{-0.400t}$	0.998	0.400	1.73
[Pro][LacA]	$C_{t}=12.5e^{-0.391t}$	0.997	0.391	1.77
[Pro][PyrA]	$C_{t}=13.4e^{-0.383t}$	0.993	0.383	1.81
[Pro][NonA]	$C_{t}=10.6e^{-0.438t}$	0.994	0.438	1.58
[Pro][OleA]	$C_{t}=9.54e^{-0.552t}$	0.996	0.552	1.26
[Pro][BenA]	$C_{t}=9.35e^{-0.774t}$	0.999	0.774	0.90
[Pro][SalA]	$C_{t}=9.59e^{-0.401t}$	0.994	0.401	1.73
[Pro][CinA]	$C_{t}=11.2e^{-0.305t}$	0.991	0.305	2.27

## Data Availability

Data are available upon request from the corresponding author.

## Supplementary Materials

The following supporting information can be downloaded at: https://www.mdpi.com/article/10.3390/molecules30071641/s1, Experimental section for pre-treatment of mango samples to determine prochloraz; HPLC-UV specifications; HPLC-MS/MS specifications; Table S1: Experimental parameters (MRM Mode) of HPLC-MS/MS; Table S2: ^1^H NMR data of PILs; Table S3: The photolysis kinetics of prochloraz in the mixture with sodium cinnamate; Table S4: Acute toxicity of prochloraz and PILs to zebrafish embryo; Table S5: Fungicidal activity against *Colletotrichum gloeosporioides*; Table S6: Fungicidal activity of 8 acids and their sodium salts at a concentration of 26.5 μmol/L.

## Abbreviations

ILs	Ionic liquids
PILs	Prochloraz-based ionic liquids
APIs	Active pharmaceutical ingredients
AceA	Acetic acid
LacA	Lactic acid
PyrA	Pyruvic acid
NonA	Nonanoic acid
OleA	Oleic acid
BenA	Benzoic acid
SalA	Salicylic acid
CinA	Cinnamic acid
