# Supplementary material for: Enhancing Photostability of Prochloraz via Designing Natural Acid-Derived Prochloraz-Based Ionic Liquids
Source: Molecules. 2025 Apr 7;30(7):1641. doi: 10.3390/molecules30071641 (PMC11990615; doi:10.3390/molecules30071641)
Supplement: Supplementary file 1 [file molecules-30-01641-s001.zip › molecules-3536732-supplementary.pdf]

Supporting Information

Enhancing Photostability of Prochloraz via Designing Natural Acid-Derived Prochloraz Ionic Liquids

Zhiqiang Gao[1], Fengmao Liu[1], Qingrong Peng[1], Wenzhuo Wang*

Affiliation 1: Innovation Center of Pesticide Research, Department of Applied Chemistry, College of Science, China Agricultural University, Beijing 100193, China

* Corresponding author:

Fengmao Liu − Innovation Center of Pesticide Research, Department of Applied Chemistry, College of Science, China Agricultural University, Beijing 100193, China; orcid.org/0000-0002-1958-7230; Phone: 0086-10-62731978; Email: *lfm2000@cau.edu.cn;* Fax: +0086-10-62733620.

Experimental Section

Pre-treatment of mango samples to determine prochloraz. Five grams of homogenized flesh (or peel) of mangos was accurately weighed into a 50 mL Teflon centrifuge tube and 10 mL of acetonitrile was subsequently added to the tube to extract the target compounds. The mixture was then vortexed for 5 min using a Multi-Tube Vortexer, after which the tube was centrifuged for 5 min at 3800 rpm. After centrifugation, 1 mL of the supernatant was added to a 2 mL microcentrifuge tube containing 50 mg PSA (primary secondary amine). The mixture was vortexed for 2 min to ensure sufficient contact between the purifying agents and the nontarget compounds, and then centrifuged at 10,000 rpm for 1 min. The supernatant was filtered through a 0.22 μm membrane and transferred into an autosampler vial using a syringe for HPLC-MS/MS analysis.

Determination of the purity of products. The purity of the synthesized ionic liquids was determined via high-performance liquid chromatography (HPLC). Precisely 0.0500 g of the products was weighed, respectively, and dissolved in methanol within a 50 mL volumetric flask, then diluted to the mark. The resulting solution was diluted tenfold with methanol to prepare the test sample. Quantification was performed using the external standard method in liquid chromatography to calculate the product purity.

HPLC-UV specifications. The chromatographic analysis was conducted using an Agilent 1260 Infinity II HPLC system equipped with a UV detector. Separation was performed on an Agilent XDB-C18 column (4.6 × 250 mm, 5 μm particle size) using a mobile phase consisting of acetonitrile and 0.1% formic acid in water (85:15, v/v) at a flow rate of 1.0 mL/min. Detection was set at a wavelength of 220 nm.

HPLC-MS/MS specifications. Chromatographic analysis was conducted using an Agilent 6410A high-performance liquid chromatograph coupled with a triple quadrupole mass spectrometer. The separation of prochloraz was achieved using a C18 column (2.1 × 50 mm, 3 μm particle size). The isocratic mobile phase consisted of acetonitrile and water (0.1% formic acid) in an 85:15 (v/v) ratio. The flow rate was set at 0.3 mL/min, and the sample injection volume was 5 μL.

The target compounds were analyzed with a triple-quadrupole mass spectrometer equipped with an ESI source in positive ionization mode. Multiple reaction monitoring (MRM) mode was employed for the analysis. Specific details are provided in Table S1.

Table S1. Experimental parameters (MRM Mode) of HPLC-MS/MS.

Compound	Retention time (min)	Qualitative ion (m/z)	Quantitative ion (m/z)	Fragmentor (eV)	Collision energy (eV)
Prochloraz	0.92	376.0→308.0	376.0→308.0	80	8
		376.0→266.0		80	12

Table S2. ^{1}H NMR data of PILs.

Chemicals	δ /ppm	Purity /%
Prochloraz	7.94 (s, 1H), 7.30 (d, J = 13.4 Hz, 3H), 7.10 (s, 1H), 4.21 (t, J = 5.1 Hz, 2H), 3.86 (t, J = 5.0 Hz, 2H), 3.57 – 3.51 (m, 2H), 1.73 (h, J = 7.5 Hz, 2H), 0.92 (t, J = 7.4 Hz, 3H).	99
[Pro][AceA]	8.01 (s, 1H), 7.30 (d, J = 7.6 Hz, 3H), 7.12 (s, 1H), 4.20 (t, J = 5.0 Hz, 2H), 3.85 (t, J = 5.0 Hz, 2H), 3.56 – 3.50 (m, 2H), 2.07 (s, 3H), 1.73 (h, J = 7.5 Hz, 2H), 0.91 (t, J = 7.4 Hz, 3H).	99
[Pro][LacA]	8.09 (s, 1H), 7.31 (s, 3H), 7.14 (t, J = 1.1 Hz, 1H), 5.77 (s, 2H), 4.32 (dq, J = 38.2, 7.0 Hz, 1H), 4.20 (t, J = 5.0 Hz, 2H), 3.85 (t, J = 5.0 Hz, 2H), 3.57 – 3.49 (m, 2H), 1.74 (p, J = 7.5 Hz, 2H), 1.62 – 1.42 (m, 3H), 0.92 (t, J = 7.4 Hz, 3H).	96
[Pro][PyrA]	12.06 (s, 1H), 8.27 (s, 1H), 7.36 (s, 1H), 7.31 (s, 2H), 7.22 (s, 1H), 4.19 (t, J = 5.0 Hz, 2H), 3.85 (t, J = 5.0 Hz, 2H), 3.53 (dd, J = 8.8, 6.7 Hz, 2H), 2.45 (s, 2H), 1.79 – 1.67 (m, 2H), 0.92 (t, J = 7.4 Hz, 3H).	95
[Pro][NonA]	8.01 (s, 1H), 7.30 (d, J = 8.9 Hz, 3H), 7.14 – 7.10 (m, 1H), 4.20 (t, J = 5.0 Hz, 2H), 3.85 (t, J = 5.0 Hz, 2H), 3.57 – 3.50 (m, 2H), 2.32 (t, J = 7.5 Hz, 2H), 1.73 (h, J = 7.5 Hz, 2H), 1.63 (q, J = 7.4 Hz, 2H), 1.32 (s, 2H), 1.31 – 1.23 (m, 8H), 0.89 (dt, J = 24.6, 7.2 Hz, 6H).	96
[Pro][OleA]	8.00 (s, 1H), 7.31 (d, J = 11.1 Hz, 3H), 7.13 (s, 1H), 5.39 – 5.29 (m, 2H), 4.21 (t, J = 5.0 Hz, 2H), 3.86 (t, J = 5.0 Hz, 2H), 3.57 – 3.51 (m, 2H), 2.34 (t, J = 7.5 Hz, 2H), 2.00 (q, J = 6.3 Hz, 4H), 1.73 (p, J = 7.5 Hz, 2H), 1.63 (t, J = 7.3 Hz, 2H), 1.28 (dt, J = 21.3, 6.7 Hz, 20H), 0.90 (dt, J = 23.8, 7.2 Hz, 6H).	95
[Pro][BenA]	8.11 (dd, J = 8.1, 1.5 Hz, 2H), 8.08 (s, 1H), 7.61 – 7.54 (m, 1H), 7.46 (t, J = 7.7 Hz, 2H), 7.32 (d, J = 3.1 Hz, 3H), 7.18 (d, J = 1.5 Hz, 1H), 4.21 (t, J = 5.0 Hz, 2H), 3.86 (t, J = 5.0 Hz, 2H), 3.58 – 3.51 (m, 2H), 1.75 (dt, J = 15.2, 7.5 Hz, 2H), 0.93 (t, J = 7.4 Hz, 3H).	97
[Pro][SalA]	11.83 (s, 1H), 8.22 (s, 1H), 7.93 (dd, J = 7.8, 1.8 Hz, 1H), 7.44 (ddd, J = 8.7, 7.2, 1.8 Hz, 1H), 7.37 (d, J = 1.5 Hz, 1H), 7.32 (s, 2H), 7.24 – 7.21 (m, 1H), 6.97 (dd, J = 8.4, 1.1 Hz, 1H), 6.91 – 6.85 (m, 1H), 4.22 (t, J = 5.0 Hz, 2H), 3.87 (t, J = 5.0 Hz, 2H), 3.59 – 3.52 (m, 2H), 1.77 (dt, J = 15.2, 7.5 Hz, 2H), 0.94 (t, J = 7.4 Hz, 3H).	98
[Pro][CinA]	8.05 (d, J = 1.4 Hz, 1H), 7.75 (d, J = 16.0 Hz, 1H), 7.58 – 7.50 (m, 2H), 7.38 (p, J = 3.8, 3.4 Hz, 3H), 7.31 (s, 3H), 7.15 (t, J = 1.2 Hz, 1H), 6.47 (d, J = 16.0 Hz, 1H), 4.21 (t, J = 5.0 Hz, 2H), 3.86 (t, J = 5.0 Hz, 2H), 3.58 – 3.51 (m, 2H), 1.74 (h, J = 7.5 Hz, 2H), 0.92 (t, J = 7.4 Hz, 3H).	99

^{1}H NMR (500 MHz, Chloroform- *d*)

Photostability of Prochloraz in the Mixture with Sodium Cinnamate. As a comparative photostability study with [Pro][CinA], a mixture of prochloraz and sodium cinnamate was dispersed at the corresponding concentration, with the results presented in Table S2.

Table S3. The photolysis kinetics of prochloraz in the mixture with sodium cinnamate.

Objects	Regression equation	r^2	$K(h^{-1})$	$t_{1/2}$(h)
Mixture of prochloraz and sodium cinnamate	$C_t = 18.0e^{-0.367t}$	0.993	0.367	1.89

Table S4. Acute toxicity of prochloraz and PILs to zebrafish embryo.

Chemicals	Regression equation	R^2	LC_{50}-96h		95% confidence interval	Significance (v.s. prochloraz)
			(μmol/L)	(mg/L)	(μmol/L)	
Prochloraz	y=1.68x-3.75	0.930	18.84	7.09	16.85–20.86	
[Pro][AceA]	y=1.13x-1.90	0.889	11.44	4.99	9.87–13.42	** ($t = -5.42$, $p < 0.01$)
[Pro][LacA]	y=2.15x-2.55	0.906	7.10	3.32	6.53–7.74	** ($t = -10.99$, $p < 0.01$)
[Pro][PyrA]	y=2.48x-1.01	0.943	3.78	1.76	3.51–4.09	** ($t = -14.57$, $p < 0.01$)
[Pro][NonA]	y=2.21x-4.53	0.913	15.67	8.38	14.39–17.03	n.s. ($t = -2.59$, $p > 0.05$)
[Pro][OleA]	y=2.17x-6.19	0.873	31.36	20.67	28.84–34.05	** ($t = 7.46$, $p < 0.01$)
[Pro][BenA]	y=2.16x-4.43	0.859	15.25	7.61	13.83–16.56	* ($t = -2.90$, $p < 0.05$)
[Pro][SalA]	y=1.05x-1.56	0.897	9.68	4.98	7.91–11.13	** ($t = -6.98$, $p < 0.01$)
[Pro][CinA]	y=1.19x-3.13	0.898	26.09	13.69	22.19–34.02	n.s. ($t = 2.28$, $p > 0.05$)

Significance levels: n.s. $p > 0.05$, * $p < 0.05$, ** $p < 0.01$, *** $p < 0.001$.

Table S5. Fungicidal activity against *Colletotrichum gloeosporioides.*

Chemicals	Regression equation	R2	IC_{50}		95% confidence interval	Significance (v.s. prochloraz)
			(μmol/L)	(mg/L)	(μmol/L)	
Prochloraz	y=0.64x+0.52	0.892	1.39	0.52	1.14–1.72	
[Pro][AceA]	y=0.69x+0.67	0.901	1.22	0.53	1.01–1.49	n.s. ($t = -0.89$, $p > 0.05$)
[Pro][LacA]	y=0.82x+0.42	0.975	1.74	0.81	1.60–1.89	n.s. ($t = 2.12$, $p >0.05$)
[Pro][PyrA]	y=0.69x+0.43	0.948	1.60	0.75	1.41–1.85	n.s. ($t = 1.13$, $p > 0.05$)
[Pro][NonA]	y=0.84x+0.29	0.921	1.98	1.06	1.64–2.45	n.s. ($t = 2.32$, $p > 0.05$)
[Pro][OleA]	y=0.56x+0.18	0.924	2.02	1.33	1.71–2.46	n.s. ($t = 2.60$, $p > 0.05$)
[Pro][BenA]	y=0.87x+0.23	0.954	2.09	1.04	1.87–2.37	* ($t = 3.58$, $p < 0.05$)
[Pro][SalA]	y=0.78x+0.30	0.973	1.94	1.00	1.78–2.12	* ($t = 3.21$, $p < 0.05$)
[Pro][CinA]	y=0.82x+0.27	0.968	2.00	1.05	1.84–2.19	* ($t = 3.53$, $p < 0.05$)

Significance levels: n.s. $p > 0.05$, * $p < 0.05$, ** $p < 0.01$, *** $p < 0.001$.

Table S6 Fungicidal activity of 8 acids and their sodium salts at a concentration of 26.5 μmol/L.

Chemicals	Inhibition rate	Chemicals	Inhibition rate
Prochloraz	100.0 ± 0.1%		
Acetic acids	8.3 ± 2.3%	Sodium acetate	5.9 ± 0.5%
Lactic acids	6.0 ± 1.0%	Sodium lactate	4.0 ± 0.4%
Pyruvic acids	4.5 ± 0.4%	Sodium pyruvate	3.5 ± 0.2%
Nonanoic acids	1.8 ± 1.3%	Sodium nonanoate	2.9 ± 0.3%
Oleic acids	3.4 ± 1.3%	Sodium oleate	3.9 ± 2.6%
Benzoic acids	7.2 ± 1.2%	Sodium benzoate	3.0 ± 1.1%
Salicylic acids	4.5 ± 0.3%	Sodium Salicylate	2.0 ± 2.7%
Cinnamic acids	7.5 ± 2.4%	Sodium cinnamate	2.8 ± 1.1%
